# Efficient Removal of Copper Ion from Wastewater Using a Stable Chitosan Gel Material

**DOI:** 10.3390/molecules24234205

**Published:** 2019-11-20

**Authors:** Zujin Yang, Yuxin Chai, Lihua Zeng, Zitao Gao, Jianyong Zhang, Hongbing Ji

**Affiliations:** 1 School of Chemical Engineering and Technology, Sun Yat-sen University, Zhuhai 519082, China; chaiyx@mail2.sysu.edu.cn (Y.C.); gaozt@mail2.sysu.edu.cn (Z.G.); 2 Huizhou Research Institute of Sun Yat-sen University, Huizhou 516216, China; 3 School of Materials Science and Engineering, MOE Laboratory of Polymeric Composite and Functional Materials, Sun Yat-sen University, Guangzhou 510275, China; Zenglh8@mail2.sysu.edu.cn (L.Z.); zhjyong@mail.sysu.edu.cn (J.Z.); 4 Fine Chemical Industry Research Institute, The Key Laboratory of Low-carbon Chemistry & Energy Conservation of Guangdong Province, School of Chemistry, Sun Yat-sen University, Guangzhou 510275, China; 5 School of Chemical Engineering, Guangdong University of Petrochemical Technology, Maomen 525000, China

**Keywords:** functional chitosan gel, Cu^2+^ ion, adsorption, mechanism, reuse experiment

## Abstract

Gel adsorption is an efficient method for the removal of metal ion. In the present study, a functional chitosan gel material (FCG) was synthesized successfully, and its structure was detected by different physicochemical techniques. The as-prepared FCG was stable in acid and alkaline media. The as-prepared material showed excellent adsorption properties for the capture of Cu^2+^ ion from aqueous solution. The maximum adsorption capacity for the FCG was 76.4 mg/g for Cu^2+^ ion (293 K). The kinetic adsorption data fits the Langmuir isotherm, and experimental isotherm data follows the pseudo-second-order kinetic model well, suggesting that it is a monolayer and the rate-limiting step is the physical adsorption. The separation factor (*R*_L_) for Langmuir and the *1/n* value for Freundlich isotherm show that the Cu^2+^ ion is favorably adsorbed by FCG. The negative values of enthalpy (Δ*H*°) and Gibbs free energy (Δ*G*°) indicate that the adsorption process are exothermic and spontaneous in nature. Fourier transform infrared (FTIR) spectroscopy and x-ray photoelectron spectroscopy (XPS) analysis of FCG before and after adsorption further reveal that the mechanism of Cu^2+^ ion adsorption. Further desorption and reuse experiments show that FCG still retains 96% of the original adsorption following the fifth adsorption–desorption cycle. All these results indicate that FCG is a promising recyclable adsorbent for the removal of Cu^2+^ ion from aqueous solution.

## 1. Introduction

Water pollution caused by heavy metal ion poses a significant threat to environment and human health due to its non-degradability and toxicity [1,2]. Cu^2+^ ion usually exists in wastewaters of many industries including mining, metallurgical, printed circuits, pipe corrosion, and metal plating industries, etc. [3,4]. However, improper disposal of wastewaters containing Cu^2+^ ion can bring human health issues and serious environmental problems. According to the U.S. Environmental Protection Agency (EPA), the maximum acceptable concentration of Cu^2+^ ion in drinking water is 1.3 mg/L [5]. An excessive amount of Cu^2+^ ion in the human body could cause serious harm to the kidneys or liver [6,7]. Therefore, developing an efficient adsorbent is necessary to remove Cu^2+^ ion from aqueous solution. Many techniques have been used for the removal of Cu^2+^ ion from various industrial effluents, such as chemical precipitation, ion exchange, membrane filtration, and adsorption [8,9,10]. Among these methods, biosorption offers an effective method for the removal of Cu^2+^ ion from aqueous solution because it has obvious advantages, such as good stability and selectivity, biocompatibility, and biodegradability [11,12].

Chitosan is produced by deacetylation of chitin, which is the second most abundant natural polymer in nature, after cellulose. It has unique properties among biopolymers due to the presence of the amino and hydroxyl groups, and it is extensively applied in wastewater treatment containing heavy metals [13,14]. However, its practical application has been restricted by poor stability in acid solution [15,16]. Chemical modification has been used to stabilize chitosan in acid solution by using cross-linking reagents, such as formaldehyde, glutaraldehyde, glyoxal, epichlorohydrin, ethylene glycondiglycidyl ether, and isocyanates [17,18]. The materials offer some obvious advantages, such as their chemical stability, large specific surface area, and abundant pore-size distribution compared to other adsorbents. For these characteristics, the cross-linked chitosan gels have been applied for the removal of heavy metal ions from aqueous solution [19,20,21]. However, the immobilization process during the cross-linking reaction would result in the decrease of some adsorption sites, reducing adsorption capacity. However, remaining amine groups offer a promising method to improve the adsorption of pollutants in wastewater [22,23]. Reduction to active functional groups from cross-linking groups has been rarely reported in the previous literature.

**Scheme 1:**
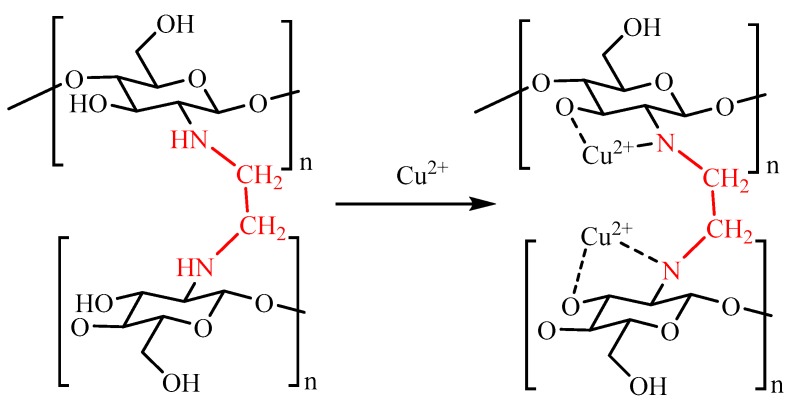
Adsorption mechanism of FCG with Cu^2+^ ion.

In the present work, we converted chitosan power into an insoluble polymer by cross-linking reaction with glyoxal (Gly), which is a promising cross-linker because of its desirable properties [24]. The obtained polymer was further reduced with sodium borohydride (NaBH_4_) to obtain functional chitosan gel material (FCG) (Scheme 1). The resulting FCG was analyzed by Brunau–Emmet–Teller (BET) analysis, Fourier transform infrared spectra (FTIR), solid-state nuclear magnetic resonance (NMR), scanning electronic microscope (SEM), thermogravimetric analysis (TGA), and X-ray photoelectron spectroscopy (XPS). The effects of pH, contact time, ionic strength, and temperature on the adsorption capacity are tested, and the adsorption properties of FCG are also evaluated, using Cu^2+^ ion as the model ion. Finally, the adsorption mechanism is discussed, according to the XPS and FTIR analyses.

## 2. Results and Discussion

### 2.1. Characterization of FCG

**Figure 1:**
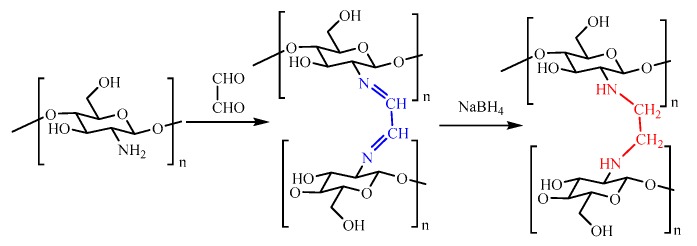
The synthesis procedure of functional chitosan gel (FCG).

As displayed in Figure 1, FCG gel was obtained by reacting chitosan with glyoxal in acetic acid aqueous solution. The obtained polymer was further reduced with NaBH_4_ saturated solution to obtain a white three-dimensional porous gel. For comparison, chitosan and FCG were soaked in distilled water, 2% acetic acid, or NaOH (0.1 mol/L) for 1 day, at room temperature (RT). As shown in Table S1, there were no significant changes for FCG, indicating that it is stable in acidic or basic solution at RT. Thus, the stability of FCG is due to the formation of C-N covalent bonding. In addition, the low swelling behavior of FCG demonstrates that it can be applied for the adsorption experiments.

Figure S1 shows N_2_ adsorption–desorption isotherms at 77 K and pore-size distribution for chitosan and FCG. As shown in Table S2, the specific surface area of FCG (2.53 m^2^/g) is much higher than that of chitosan (0.58 m^2^/g). The average pore diameter and total pore volume of chitosan and FCG are 19.88 nm, 0.00269 cm^3^/g, and 4.63 nm, 0.0829 cm^3^/g, respectively. It can be seen that the surface area and total pore volume increase, but the average pore diameter decrease, after reaction, implying that the synthesized FCG is favorable for the adsorption of metal ions. Based on the IUPAC results, N_2_ adsorption of FCG belongs to type II. It exhibits a significant rise of uptake at the relative pressure (P/P° = 0 to 0.05), and steadily rises within the region 0.1 < P/P° < 0.9 and rapidly climbs in the range of P/P° > 0.9, demonstrating that the FCG is micropores [25]. The results indicate that the improved porosity is beneficial for the penetration of Cu^2+^ ion into the network of FCG.

**Figure 2:**
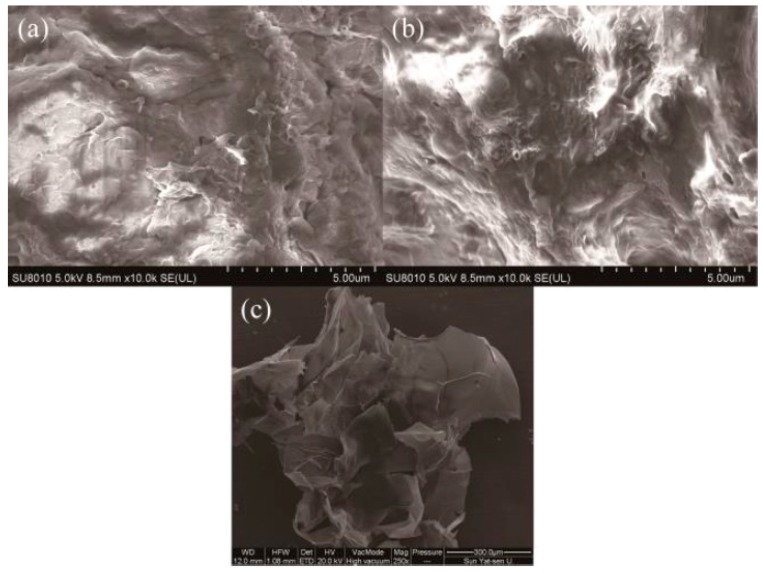
SEM micrographs of chitosan (**a**), FCG, (**b**) and FCG after adsorption (**c**).

As Figure 2a shows, chitosan has a smooth and heterogeneous surface with micropore structure, which has been confirmed by its BET surface area. FCG has a much more wrinkled and irregular structure than non-cross-linked chitosan, and large pore structures can be clearly seen in Figure 2b. However, after adsorption of Cu^2+^ ion, the strong and specific binding of Cu^2+^ involving most of the –NH and –OH groups lead to a smooth surface structure (Figure 2c). In addition, FCG still retains their original connected three-dimensional structure, verifying its repeatability.

**Figure 3:**
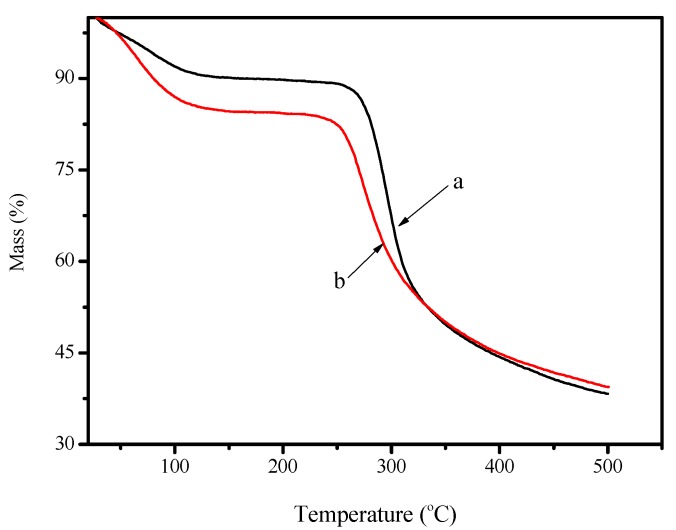
TGA curves of chitosan (**a**) and FCG (**b**).

Figure 3 shows TG curves of chitosan and FCG. The first mass-loss stages from about 323 to 393 K are due to the loss of water adsorbed on the surface of the adsorbents. The second stage begins at 553 K with mass loss of 65% due to heat decomposition of chitosan. The TGA curve for FCG exhibits that thermal degradation starts at 393 K with 42% mass loss, which is lower than that of chitosan. The result implies that FCG is less stable than chitosan, which may be due to a decrease of intramolecular hydrogen bonding. However, the thermal stability of FCG is sufficient for the material to be used in wastewater treatment.

**Figure 4:**
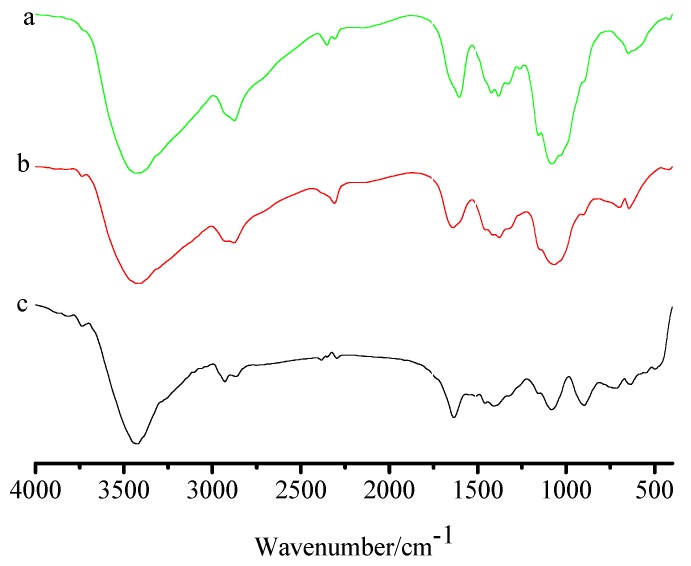
FTIR spectra of chitosan (**a**), FCG (**b**), and FCG after adsorption of Cu^2+^ ion (**c**).

Figure 4 shows the FTIR spectra of chitosan, FCG (**b**), and FCG after adsorption of Cu^2+^ ion (**c**). the bands of 3430 cm^−1^ (–OH stretching vibration), 1570 cm^−1^ (N–H bending vibration of –NH_2_ groups), and 1032 cm^−1^ (O–H bending vibration) represent the characteristic absorption peak of chitosan (Figure 4a) [26]. After it reacts with Gly and is further reduced by NaBH_4_, the N–H bending vibration of chitosan at 1570 cm^−1^ is obviously weakened, and a new peak is observed at 1640 cm^-1^, due to the formation of –NH–CH_2_ group. The presence of the –NH–CH_2_ group is confirmed by C–H stretching vibration at 2927 cm^−1^. The results indicate that Gly monomer is grafted onto the backbone of chitosan (Figure 4b). In order to further illustrate the adsorption mechanism of FCG for Cu^2+^ ion, the FTIR spectrum of FCG after adsorption was recorded. Compared with the spectrum of FCG before adsorption, the NH stretching vibration of –NH–CH_2_ group at 1570 cm^−1^ was shifted to 1520 cm^−1^ and decreased in intensity after the adsorption of Cu^2+^ ion, indicating that the NH and OH groups complexed with the Cu^2+^ ion (Figure 4c).

**Figure 5:**
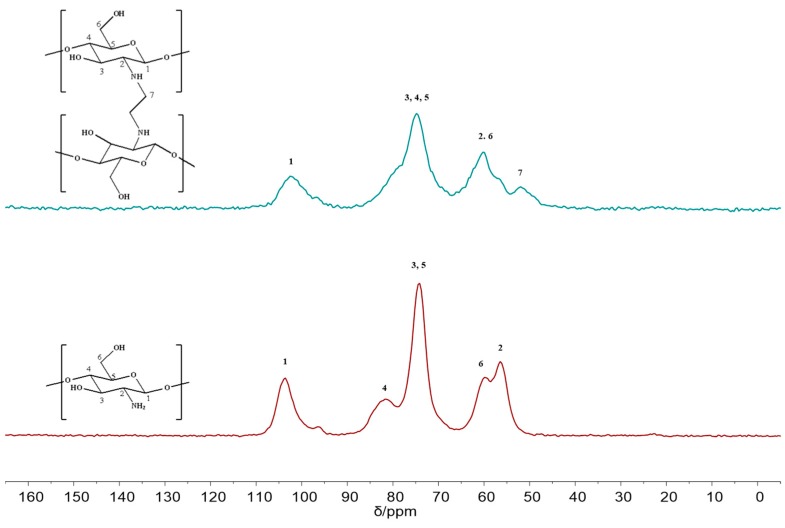
^13^C CP–MAS spectra of chitosan and FCG.

Solid-state ^13^C NMR spectra of chitosan and FCG are shown in Figure 5. The chemical shift of chitosan reveals six ^13^C signals at δ 57 ppm (C–N), 60 ppm (C2, C6), 75 ppm (C3, C4, C5), and 102 ppm (C1), respectively [27]. FCG has similar structure characteristics with chitosan. However, peak for FCG at δ 54 ppm implies the formation of C–N bond (C7) [28,29].

**Figure 6:**
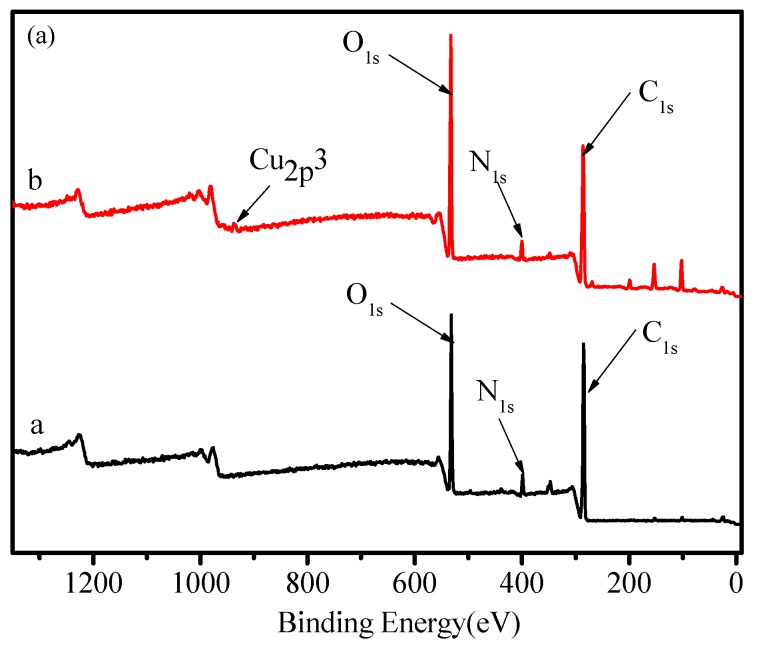

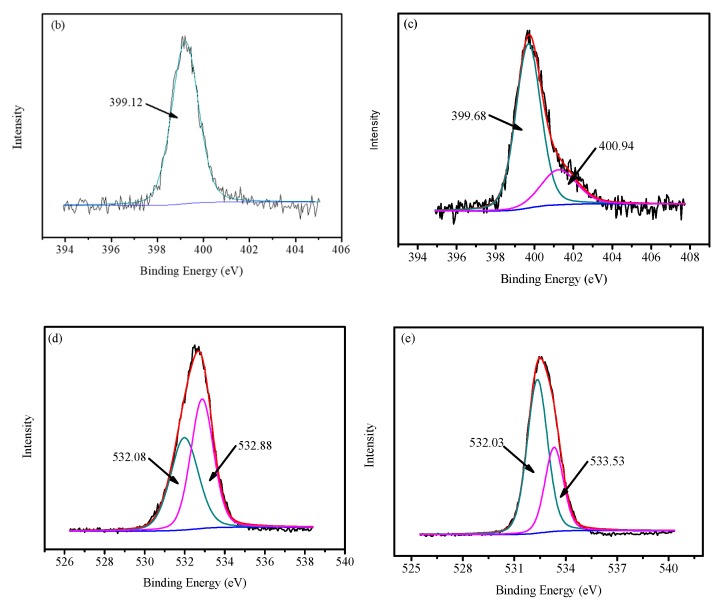
XPS spectra, N_1s_ and O_1s_ of FCG before (**a**,**c**) and after (**b**,**d**) adsorption of Cu^2+.^ ion.

Figure 6 shows the XPS spectra of FCG before and after the adsorption. It is obvious that the Cu_2p_ (*BE* = 933.53 eV) peak appeared in the spectra of the FCG after the adsorption, implying that the Cu^2+^ ion was adsorbed on the FCG (Figure 6a). The N1s exhibits a single peak with the bonding energy of 399.12 eV (the N atom in the NH group, Figure 6b), slightly shifted to 399.68 eV after the adsorption and a new peak appears at 400.94 eV due to the N1s BE of NH2-C groups in the complex compounds of Cu^2+^ ion with FCG, confirming that the nitrogen atom of –NH groups could also form surface complexes with Cu^2+^ ion (Figure 6c). The O1s peaks at 532.08 eV (C–O) and 532.88 eV (C–OH) for the FCG (Figure 6d) shifted to 532.02 (C–O) and 533.53 (C–OH) (Figure 6e), respectively, after Cu^2+^ ion adsorption. The results indicate that the –NH and –OH groups complexed with Cu^2+^ ion, and a chemical adsorption process occurred (Scheme 1).

### 2.2. Batch Adsorption Experiment

#### 2.2.1. Effect of pH of the Solution

**Figure 7:**
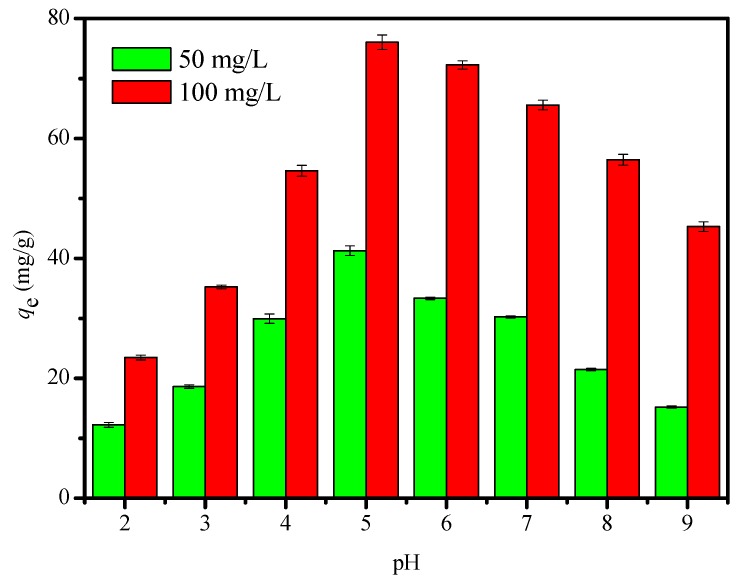
Effect of the solution pH on the adsorption of Cu^2+^ ion. (Initial Cu^2+^ ion concentration, 50 and 100 mg/L; temperature, 293 K; pH 7; agitation speed, 150 r/min; and adsorbent dose, 10 mg/25 mL.)

The adsorption of metal ion is significantly affected by pH of the solution, which may affect the surface charge between adsorbent and adsorbate at different pH value [30,31]. Figure 7 shows the effect of pH on the adsorption of Cu^2+^ ion for FCG in the pH range of 2–9. Initially, adsorption quantity of FCG toward Cu^2+^ ion increases quickly, and, after some time, the adsorption process of Cu^2+^ gradually reaches equilibrium. The maximum adsorption capacities are 76.03 and 41.25 mg/g when the initial concentrations of Cu^2+^ ion are 100 and 50 mg/L at pH = 5, respectively. Cu^2+^ exists in different anion species, such as Cu^2+^, Cu(OH) or Cu(OH)_2_, at different pH value [32]. Cu^2+^ is the main species at pH = 2–5, while Cu(OH)_2_ is the main species at pH > 5. When pH value is low, competition between protons (H^+^) and Cu^2+^ for the adsorption sites of the sorbent would decease electrostatic interaction to some extent, and this electrostatic repulsion prevents the adsorption of Cu^2+^ ion on the surface of FCG. The protonation degree of adsorbent decreases with the increase of pH to neutral, the adsorption of Cu^2+^ ion significantly increases, and maximum adsorption capacity is observed about near pH 5, which is attributed to the fact that a greater number of Cu^2+^ ions can exchange with the functional groups of FCG. Precipitation of Cu(OH)_2_ occurs between Cu^2+^ ion and hydroxyl ion (OH^−^) at pH > 5. Based on the above results, the experiments were performed at pH of 5. Similar results have been reported for the capture of metal ion at different pH values [33,34,35]. The maximum adsorption capacity of Cu^2+^ ion with the previously reported chitosan-based adsorbents are shown in the Table 1. It can be seen that FCG has a relatively large adsorption capacity, indicating that FCG is a fairly good material for the removal of Cu^2+^ ion from aqueous solution.

#### 2.2.2. Effects of Ionic Strength

**Figure 8:**
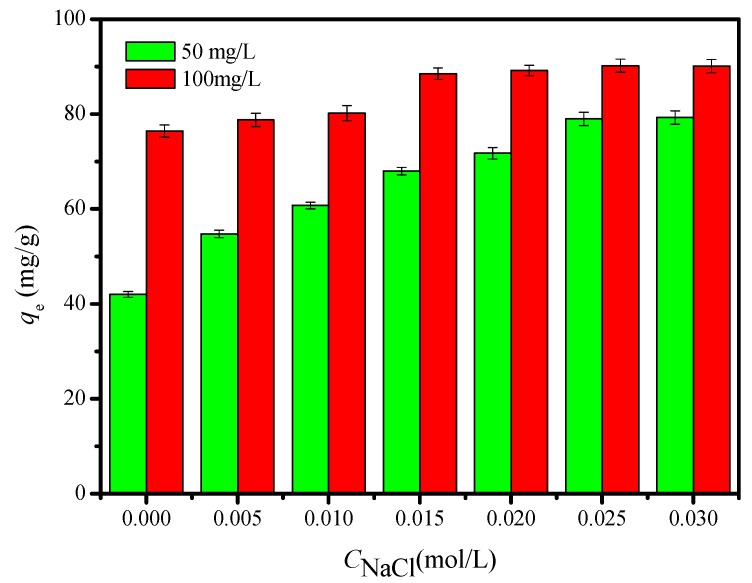
Effects of solution ionic strength on the Cu^2+^ ion adsorption by FCG. (Initial Cu^2+^ ion concentration, 50 and 100 mg/L; temperature, 293 K; pH, 5; agitation speed, 150 r/min; and adsorbent dose, 10 mg/25 mL.)

Effect of NaCl on the adsorption of Cu^2+^ ion is also tested by varying NaCl concentration from 0 to 0.030 mol/L, as shown in Figure 8. The change of NaCl concentration shows significant effects on the adsorption of Cu^2+^ ion with the different concentration. The results indicate that *q_e_* of Cu^2+^ ion increased from 54.75 and 78.80 mg/g to 79.90 and 90.02 mg/g, respectively, when the Cu^2+^ ion concentration of 50 and 100 mg/L with increasing NaCl concentration from 0 to 0.030 mol/L. The increase of Cu^2+^ ion adsorption with higher ionic strength could be attributed to rise in activity coefficient of Cu^2+^ ion, decreasing the solubility of Cu^2+^ ion and enhancing their adsorption capacities. In addition, the increase of ionic strength might hinder the ion exchange between FCG and Cu^2+^ ion [42]. There was no obvious change when the NaCl concentration exceeded 0.025 mol/L; thus, 0.025 mol/L NaCl solution is chosen in this study.

#### 2.2.3. Adsorption Kinetics

**Figure 9:**
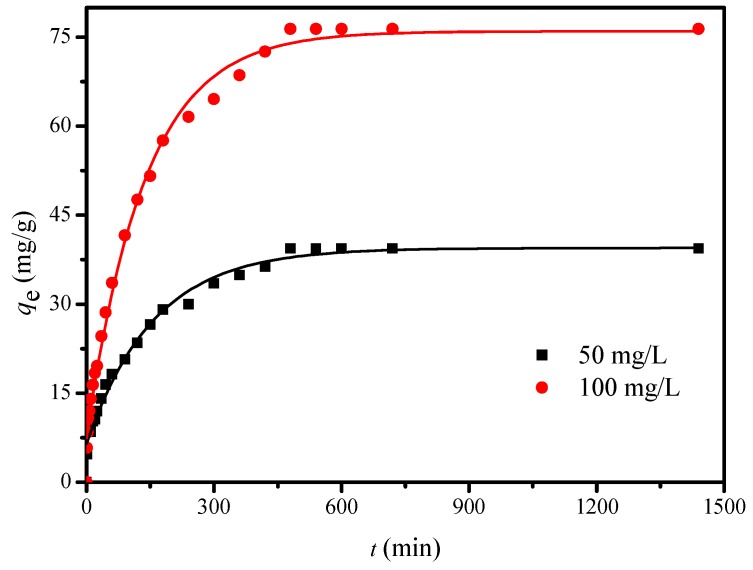
Effects of contact time on the adsorption capacity of Cu^2+^ with two Cu^2+^ concentration (initial Cu^2+^ ion concentration, 50 and 100 mg/L; pH 5; temperature, 293 K; agitation speed, 150 r/min; and adsorbent dose, 10 mg/25 mL).

The effects of contact time on the adsorption capacity of Cu^2+^ ion by FCG are shown in Figure 9. The adsorption capacity of Cu^2+^ ion climbs rapidly at the initial time of 100 min and attains high values at 500 min. When the adsorption reaches equilibrium in 750 min, the adsorption capacities are 39.40 and 76.42 mg/g when Cu^2+^ ion concentrations are 50 and 100 mg/L, respectively. It might be attributed to the fact that more binding sites are readily available at the beginning of adsorption and decrease with the prolonging time. Thus, a contact time of 750 min was chosen in this study.

The three kinetic models—the pseudo-first-order, pseudo-second-order, and intraparticle diffusion models—are used to fit the adsorption kinetic data for the two Cu^2+^ ions, and the equations are displayed in Supplementary information. The results are shown in Figure S2, and the corresponding parameters of the kinetic models are listed in Table S3. It can be seen from Table S3 that the adsorption of Cu^2+^ ion with different Cu^2+^ concentration onto FCG fits the pseudo-second-order model best based on its higher correlation coefficients *R*^2^ (>0.99). The *R*^2^ for the intraparticle diffusion model was also lower than those of the pseudo-second-order equation. In addition, the *q*_e,calcd_ values obtained from the pseudo-second-order are consistent with the *q*_e,exptl_ values, implying that chemical adsorption is involved in the rate-limiting step of the adsorption for FCG [43]. The intraparticle diffusion model indicates three fitted linears for each concentration of Cu^2+^ ion, but all the straight lines have not passed through the origin, implying that Cu^2+^ ion adsorption is not mainly controlled by into-particle adsorption process. Adsorption of Cu^2+^ ion onto FCG is divided into three steps: diffusion of Cu^2+^ ion to the external surface of FCG; diffusion of Cu^2+^ ion into the pores of FCG; and adsorption of Cu^2+^ ion on the active sites of FCG. Similar kinetic results have been published for the adsorption of different pollutants [33,44,45,46].

#### 2.2.4. Adsorption Isotherm

**Figure 10:**
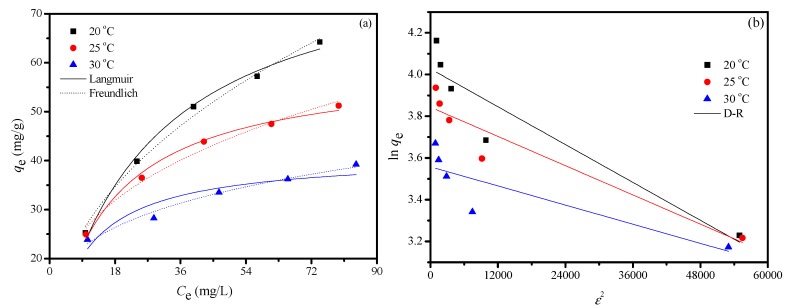
Isotherm model curves for the Cu^2+^ ion removal by the FCG; Langmuir and Freundlich model (**a**) and D-R model (**b**). (Initial Cu^2+^ concentration, 100 mg/L; pH, 5; temperature, 293 K; agitation speed, 150 r/min; and adsorbent dose, 10 mg/25 mL.).

The equilibrium adsorption of Cu^2+^ ion on the FCG as a function of the initial concentration of Cu^2+^ ion is shown in Figure 10. The adsorption capacities of Cu^2+^ ion significantly increases with increasing the initial concentration of Cu^2+^ ion, and then the increasing trend becomes slower, and adsorption almost reaches a maximum adsorption value gradually. Langmuir, Freundlich, and D-R models are used to analyze the equilibrium adsorption data of Cu^2+^ ion on the FCG, and these models are displayed in Supplementary Information.

Adsorption isotherms of Cu^2+^ ion onto FCG are shown in Figure 10, and the corresponding parameters are listed in Table S4. The Langmuir model can better describe the experimental data (Table S4) because of higher correlation coefficients (>0.99), and the maximum adsorption capacity of FCG for Cu^2+^ ion is calculated as 84.39 mg/g, based on the Langmuir model. Separation factor, *R_L_*, is widely used to describe the degree of suitability of adsorbent toward heavy metal ion, which can be expressed as follows:

(1) $$R_{L}=\frac{1}{1+K_{L}C_{O}}$$

where *C*_o_ is the initial concentration of ***Cu^2+^*** (mg/L) and *K*_L_ is the Langmuir constant. The separation factor *R*_L_ shows the type of isotherm: irreversible (*R*_L_ = 0), favorable (0 < *R*_L_ < 1), linear (*R*_L_ = 1), or unfavorable (*R*_L_ > 1) [47]. The values of *R*_L_ for Cu^2+^ ion adsorption onto FCG at the studied temperatures are all in the range of 0 < *R*_L_ < 1 (Table S4), implying that the capture of Cu^2+^ ion onto FCG is favorable [48]. The value of *R*_L_ decreases with increasing temperature, demonstrating an decrease of the affinity between Cu^2+^ ion and FCG. In addition, the values of the Freundlich adsorption isotherm constant 1/*n* are 0.46, 0.35, and 0.24, respectively. All the values are in the range of 0 to 1, implying the adsorption process is favorable. *K_f_* values for all the temperatures are found to increase with increasing temperatures, confirming the endothermic nature of adsorption. The removal of toxic heavy metal ion, such as cadmium (II), has also been tested, and the maximum adsorption capacity of FCG for cadmium is 48.02 mg/g under the similar experimental condition. The adsorption capacity is compared with the previous adsorbents, and the comparisons are displayed in Table 2. The results indicate that FCG is also an efficient adsorbent for cadmium removal from water.

#### 2.2.5. Thermodynamic Study

The calculated thermodynamic parameters of FCG are listed in Table S5. Temperature is an important factor, which affects the adsorption process of Cu^2+^ ion. The Cu^2+^ ion adsorption onto FCG was studied in the range of 293 to 303 K (Figure 10a). The results indicate that *q_m_* and *K_L_* decrease with increasing temperatures, implying that the exothermic nature of the adsorption process. The negative Δ*G*° values demonstrate the favorable and spontaneous nature of Cu^2+^ ion adsorption. The increase of Δ*G*° with the rising temperature implies that the adsorption is more beneficial at lower temperatures. The negative Δ*S*° values show the randomness at the FCG-solution interface during the adsorption. The negative Δ*H*° for the removal of Cu^2+^ ion shows the exothermic nature of adsorption process.

#### 2.2.6. Regeneration Tests

As shown in Figure S3, the adsorption capacity and recovery ratio slightly decrease with increasing the adsorption–desorption cycles. However, adsorption capacity and recovery ratio are still 72.38 mg/g and 96%, respectively, after regeneration for five times. The results show that FCG has good adsorption capacity and recovery ratio for Cu^2+^ ion.

## 3. Materials and Methods

### 3.1. Adsorbent

Chitosan with average molecular weight 200,000 and 95% deacetylation degree was obtained from Nantong Xincheng Biological Industrial Limited Co., Ltd., Nantong, China. Glyoxal (Gly) solution was supplied from Sigma-Aldrich (Sigma–Aldrich Chemie, Steinheim, Germany). The test material solution was prepared by dilution of Cu(NO_3_)_2_. Sodium borohydride (NaBH_4_), hydrochloric acid (HCl), sodium hydroxide (NaOH), and other reagents used in this work were of analytical grade and used without further purification unless indicated. Distilled water was used throughout.

### 3.2. Synthesis of FCG

Ten milligrams of chitosan were dissolved in 1 mL water containing 10 μL acetic acid, and 60 μL glyoxal was added slowly into a three-necked flask. The mixture was stirred magnetically at 298 K for 1–3 min, and 333 K for 3 h. After the aging for 12 h, the obtained product was soaked in 2 mL of saturated solution of NaBH_4_ for 72 h. After the reaction, the mixture was filtered and then washed thoroughly with large amounts of water. The resulting gel was frozen at 203 K for 48 h in a freeze-dryer.

### 3.3. Characterization of FCG

FTIR spectra were obtained on a Bruker TENSOR 37 (Bruker, Ettlingen, Germany), with the wavenumber ranging from 4000 to 400 cm^−1^ and resolution of 4 cm^−1^. The surface morphology was characterized by field-emission scanning electron microscopy (SEM) (JEOL JSM-6700F, JEOL, Ltd., Tokyo, Japan). TG experiments were carried out on a Netzsch STA-449C thermal analysis system (Netzsch Corporation, Selb, Germany). The samples of about 3 mg were heated at a heating rate of 10 K/min from room temperature to 773 K under N_2_ atmosphere. The X-ray photoelectron spectroscopy (XPS) (ESCALAB 250, Thermo Electron, Altrincham, UK) was performed with a monochromatic Al*K*α radiation source and a hemisphere detector with an energy resolution of 0.1 eV. All core-level spectra were referenced to the C1s neutral carbon peak at 284.5 eV and obtained at a take-off 90° to the sample surface. The specific surface area was determined by N_2_ adsorption isotherm at 203 K, using an ASAP 2010 Micromeritics instrument and by Brunauer−Emmett−Teller (BET) method (Micromeritics Instrument Corporation, Norcross, GA, USA).

### 3.4. Adsorption Experiments of Cu^2+^ Ion

The adsorption of Cu^2+^ ion was performed at controlled pH and temperatures (293, 298, and 303 K, respectively) by shaking 0.01 g of dry FCG with 25 mL (50 and 100 mg/L) of Cu^2+^ ion solution for 24 h at 200 r/min. The pH of the Cu^2+^ ion solution was adjusted to the desired value with HCl and NaOH. After the experiment, FCG was separated by centrifugation, and the initial and final Cu^2+^ ion concentration was determined with a Perkin-Elmer Analyst 700 atomic absorption spectrophotometer (AAS, Norwalk, CT, USA). The reproducibility for the data was within 5%.

The adsorption capacity of FCG for Cu^2+^ ion at equilibrium (*q_e_*, mg/g) and the amount of Cu^2+^ ion adsorbed per unit mass of adsorbent at time *t* (*q_t_*, mg/g) were calculated as follows:

(2) $$q_{e}=\frac{\left(C_{O}\;-\;C_{e}\right)}{W}V_{O}$$

(3) $$q_{t}=\frac{\left(C_{O}\;-\;C_{t}\right)}{W}V_{O}$$

where *C*_o_, *C_t_*, and *C*_e_ are the initial concentration, the concentration at time *t*, and the equilibrium concentration in the solution (mg/L), respectively; *V*_o_ is the volume of the initial solution (mL); and *W* is the weight of the dry FCG (g).

The effect of pH 3–9, ionic strength (0–0.030 mol/L), temperatures (293, 298, 303 K), and contact time on the adsorption capacities of Cu^2+^ ion were also studied.

### 3.5. Kinetic Experiments

Batch kinetic tests were performed by mixing 10 mg of FCG with 100 mL of Cu^2+^ ion aqueous solution with concentration of 50 and 100 mg/L with original pH of the solution at 200 r/min for the predetermined time. Then, the temperature of the mixture was kept at 293 K in a bath at constant temperature for 24 h of shaking. After adsorption, 0.5 mL of aliquots was taken at various time intervals, filtered through a 0.22 μm membrane, and diluted by using water. The residual Cu^2+^ ion concentration in the filtrate was analyzed by AAS, and the adsorption capacity was calculated by using Equation (3).

### 3.6. Adsorption Isotherm

About 10 mg of FCG was mixed with 25 mL of Cu^2+^ ion solution. The initial concentration of Cu^2+^ ion was 10–100 mg/L. The mixture was then shaken for 24 h, at a desired temperature (293, 298, and 303 K), at pH 5, until equilibrium was reached. Equilibrium concentration of Cu^2+^ ion was analyzed, and the equilibrium adsorption capacity was determined as Equation (3).

## 4. Conclusions

In summary, a functional chitosan gel (FCG) was successfully synthesized. FCG is stable at RT in acidic or basic solution, in contrast to most supramolecular gels, due to the formation of C–N covalent bonding. FCG is effective in the removal of Cu^2+^ ion from aqueous solution, and maximum adsorption capacity was 75.40 mg/g in the initial concentration of 100 mg/L. Kinetic studies indicated that the adsorption reaction follows the pseudo-second-order kinetics; this suggests the main adsorption mechanism of chemical adsorption. The adsorption isotherms could be well fitted by the Langmuir isotherm equation. Thermodynamic parameters obtained indicated that the adsorption process is spontaneous and exothermic. After regenerating five times, the adsorption capacity was 72.38 mg/g, and the respective recovery ratio reached 96%. FTIR and XPS analysis revealed that the strong electrostatic interaction between the –NH and –OH groups and Cu^2+^ ion are the main adsorption mechanisms.

## Figures and Tables

**Table 1:** Maximum adsorption capacity (mg/g) of different chitosan-based adsorbents for Cu^2+^ ion.

Adsorbent	Adsorption Capacity (mg/g)	Refs.
Thiourea-modified chitosan microspheres	66.70	[11]
Chitosan/PVA beads	47.90	[36]
Carboxymethyl chitosan-graft-d-glucuronic acid membranes	70.21	[37]
Chitosan acetate crown ether	23.90	[38]
Chitosan/cellulose composite	53.50	[39]
Glutaraldehyde-chitosan	59.61	[40]
Chitosan/SiO_2_/Fe_3_O_4_	31.72	[41]
FCG	75.40	This work

**Table 2:** Maximum adsorption capacity (mg/g) of different adsorbents for cadmium.

Adsorbent	Adsorption Capacity (mg/g)	Refs.
Bamboo charcoal	12.08	[49]
Red mud	17.80	[50]
Waste sludge	15.70	[51]
Almond shell	3.18	[52]
FCG	48.02	This work

## Supplementary Materials

The following are available online. Adsorption model fitting; Figure S1. Adsorption–desorption isotherms of N2 at 77 K and pore-size distribution of chitosan and FCG; Figure S2. Adsorption kinetic equations fitting of Cu^2+^ ions with two Cu^2+^ ions concentrations by FCG; Figure S3. Regeneration capacityand recovery ratio of FCG for Cu^2+^ ion; Table S1. Solubility and swelling of chitosan and FCG; Table S2. Porosity and diameter of chitosan and FCG; Table S3. Kinetic parameters for the adsorption of two Cu^2+^ ions concentrations at 293 K; Table S4. The correlated parameters for the adsorption of Cu^2+^ ions onto FCG from aqueous solution according to Langmuir, Freundlich and D-R models; Table S5. Thermodynamic parameters for adsorption of Cu^2+^ ions on FCG.
