# Supplementary material for: Efficient Removal of Copper Ion from Wastewater Using a Stable Chitosan Gel Material
Source: Molecules. 2019 Nov 20;24(23):4205. doi: 10.3390/molecules24234205 (PMC6930644; doi:10.3390/molecules24234205)
Supplement: Supplementary file 1 [file molecules-24-04205-s001.pdf]

Supplementary Information

Efficient Removal of Copper Ions from Wastewater Using a Stable Chitosan Gel Material

Zujin Yang,[1,4*] Yuxin Chai[1,4], Lihua Zeng[2], Zitao Gao[1], Jianyong Zhang[2], Hongbing Ji[3,4,5*]

[1]School of Chemical Engineering and Technology, Sun Yat-sen University, Zhuhai 519082, China

[2]School of Materials Science and Engineering, School of Chemistry, MOE Laboratory of Polymeric Composite and Functional Materials, Sun Yat-sen University, Guangzhou 510275, China

[3]Fine Chemical Industry Research Institute, The Key Laboratory of Low-carbon Chemistry & Energy Conservation of Guangdong Province, School of Chemistry, Sun Yat-sen University, Guangzhou 510275, China

[4]Huizhou Research Institute of Sun Yat-sen University, Huizhou 516216, China

[5]School of Chemical Engineering, Guangdong University of Petrochemical Technology, Maomen 525000, China

* Correspondence author.

Professor H. B. Ji

School of Chemistry

Sun Yat-sen University

Tel.: +86 20 84113658

No. 135, Xingang West Road

Guangzhou, China, 510275

E-mail: yangzj3@mail.sysu.edu.cn, jihb@mail.sysu.edu.cn.

Adsorption Model Fitting

In order to elucidate the adsorption mechanism, the pseudo-first-order and pseudo-second-order kinetic equations are used to fit the experimental adsorption data [**1, 2**].

$$\ln(q_e - q_t) = \ln q_e - k_1 t \quad (1)$$

$$\frac{t}{q_t} = \frac{1}{k_2 q_e^2} + \frac{t}{q_e} \quad (2)$$

In addition, the adsorption amount q_t versus $t^{0.5}$ can also be used by using the following equation [**3**],

$$q_t = k_{id} t^{0.5} + C \quad (3)$$

where q_e and q_t are the amount of Cu^{2+} ions adsorbed at equilibrium and time t (mg/g), respectively. k_1 (min^{-1}) and k_2 (mg/(g.min)) are the rate constants of pseudo-first-order and pseudo-second-order adsorption models, which can be determined by the slope and intercept from the straight lines of ln (q_e − q_t) against t and t/q_t versus t, respectively. k_{id} (mg/(g. $min^{0.5}$)) is the diffusion kinetic rate constant, which is calculated by the slope from the straight line of q_t against $t^{0.5}$.

In order to understand the equilibrium data of adsorption from aqueous solution, Langmuir, Freundlich, and Dubinin-Radushkevich (D–R) models were used study the equilibrium data of Cu^{2+} ions on the FCG [**4**].

$$\frac{c_e}{q_e} = \frac{c_e}{q_m} + \frac{1}{K_L q_m} \quad (4)$$

$$\ln q_e = \ln K_f + \frac{1}{n} \ln c_e \quad (5)$$

where c_e (mg/L) and q_e (mg/g) are the concentration of adsorbate and adsorption capacity of the adsorbent at equilibrium time, respectively. K_L is Langmuir adsorption constant and q_m (mg/g) is the theoretical maximum adsorption capacity to form monolayer on the FCG, respectively. K_f is Freundlich constant that is an indicator of adsorption capacity and $1/n$ is the Freundlich coefficient related to the magnitude of the adsorption driving force.

To further understand adsorption mechanism, D-R isotherm is applied to illustrate the adsorption process of Cu^{2+} ions as physical or chemical adsorption. Its linear form is expressed as,

$$\ln q_e = \ln q_m - \beta \varepsilon^2 \quad (6)$$

where q_m is the theoretical saturation capacity (mg/g), β is related to mean adsorption energy (kJ/mol) , and ε (Polanyi potential) is calculated as,

$$\varepsilon = RT\ (1 + \frac{1}{c_e}) \quad (7)$$

R (J/(mol.K)) is the gas constant and T (K) is absolute temperature. q_m and β are obtained from the intercept and the slope of linear plot of ln q_e against ε^2, respectively. E_a (kJ/mol) is the mean adsorption energy, which is calculated from the β value as:

$$E_a = \frac{1}{(2\beta)^{0.5}} \quad (8)$$

If E_a is > 16 kJ/mol, the adsorption process is a chemisorption, and it is a process of ion exchange when E_a is in the range of 8 to 16 kJ mol^{-1}, while for values of E_a < 8 kJ mol^{-1}, demonstrates a physical process [**5**].

Thermodynamic parameters such as free energy ($\Delta G°$), enthalpy ($\Delta H°$) and entropy ($\Delta S°$) can be calculated as,

$$K_c = \frac{C_{Ae}}{C_e} \quad (9)$$

$$\Delta G^o = -RT \ln K_c \quad (10)$$

$$\Delta G^o = \Delta H^o - T\Delta S^o \quad (11)$$

$$\ln K_c = \frac{\Delta S^o}{R} - \frac{\Delta H^o}{RT} \quad (12)$$

where K_c is the equilibrium constant, T is the absolute temperature, R is the gas constant (8.314 **J/(mol.K)**, C_e is the equilibrium concentration in solution (mg/L), C_{Ae} is the amount of Cu^{2+} adsorbed on the FCG at equilibrium (mg/L). $\Delta G°$, $\Delta H°$, $\Delta S°$ are changes in Gibbs free energy (kJ/mol), enthalpy (kJ/mol), and entropy (kJ/(mol K)) **,** respectively. The values of $\Delta H°$ and $\Delta S°$ can be calculated from the slope and intercept by plotting ln K_c against 1/T.

Figures and Tables

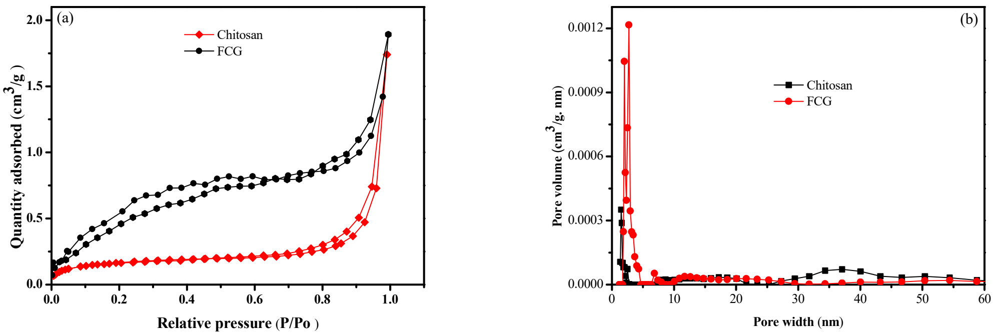

Fig. S1. (a) Adsorption–desorption isotherms of N_2 at 77 K and (b) pore-size distribution of chitosan and FCG.

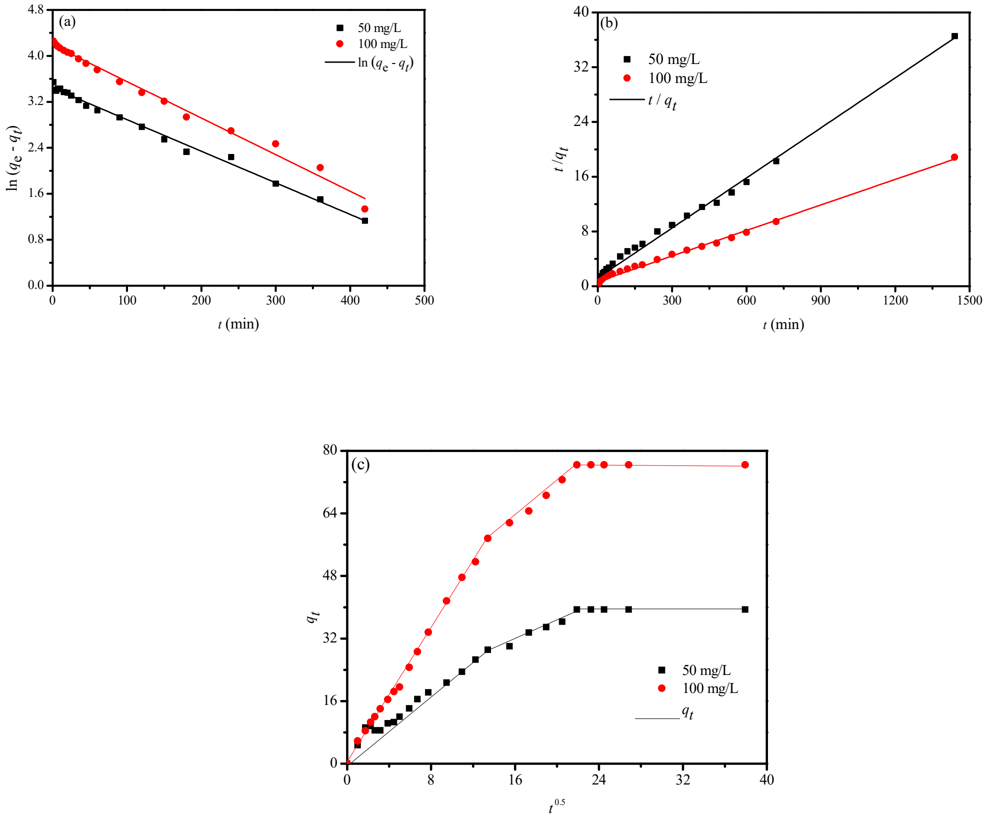

Fig.S2. Adsorption kinetic equations fitting of Cu^{2+} ions with two Cu^{2+} ions concentrations by FCG. (a) Pseudo-first-order kinetics; (b)Pseudo-second-order kinetics; (c) Intraparticle diffusion kinetics. (initial Cu^{2+} ions concentration, 50 and 100 mg/L; pH, 5; temperature, 293 K; agitation speed, 150 r/min; and adsorbent dose,10 mg/25 mL)

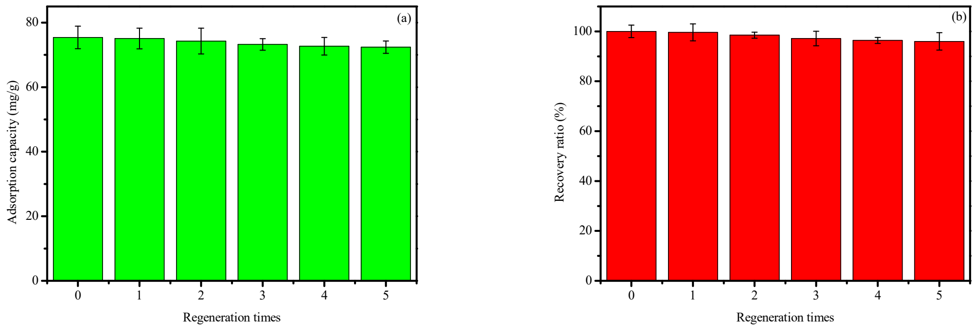

Fig. S3. Regeneration capacity (a) and recovery ratio (b) of FCG for Cu^{2+} ion.

Table S1. Solubility and swelling of chitosan and FCG

Sample	Solubility			Swelling		
	Distilled water	2% acetic acid	0.10 mol/L NaOH	Distilled water	2% acetic acid	0.10 mol/L NaOH
Chitosan	Insoluble	Soluble	Insoluble	42.4	Soluble	32.7
FCG	Insoluble	Insoluble	Insoluble	10.4	5.8	4.2

Table S2. Porosity and diameter of chitosan and FCG

Materials	Surface Area (m^2/g)	Pore Volumn (cm^3/g)	Average pore size (nm)
Chitosan	0.58	0.00269	19.88
FCG	2.53	0.0829	4.63

Table S3. Kinetic parameters for the adsorption of two Cu^{2+} ions concentrations at 293 K

model	parameter	$C_{Cu^{2+}}$（mg/L）	
		50	100
	$q_{e,exptl}$(mg/g)	39.4	76.4
Pseudo-first-order	k_1 (min^{-1})	0.0072	0.0095
	$q_{e,calcd}$(mg/g)	30.9	66.3
	R^2	0.985	0.981
Pseudo-second-order	k_2(mg/(g.min))	0.000560	0.000282
	$q_{e,calcd}$(mg/g)	39.1	77.2
	R^2	0.995	0.996
Intraparticle diffusion	k_{int}(mg/(g. min$^{0.5}$)	1.647	3.581
	C	4.656	3.761
	R^2	0.984	0.979

Table S4. The correlated parameters for the adsorption of Cu^{2+} ions onto FCG from aqueous solution according to Langmuir, Freundlich and D-R models

parameter	Temperature/**K**		
	293	298	303
	Langmuir Model		
q_m (mg/g)	84.39	60.57	43.84
K_l (L/mg)	0.07992	0.06377	0.03958
R_L	0.2016	0.1355	0.1112
R^2	0.993	0.997	0.986
	Freundlich Model		
K_f (L/mg)	13.26	11.45	8.93
$1/n$	0.46	0.35	0.24
R^2	0.992	0.984	0.971
	D-R Model		
q_m (mg/g)	56.10	46.68	35.08
β	0.004064	0.003217	0.002845
E_a (kJ mol^{-1})	13.25	12.46	11.09
R^2	0.842	0.869	0.646

Table S5. Thermodynamic parameters for adsorption of Cu^{2+} ions on FCG

adsorbent	Temperature (K)	ln K_L	Δ*G*° （kJ/mol)	Δ*H*° (kJ/mol）	Δ*S*° (J/(mol· *K*))
Cu^{2+}	293	2.992	-7.288	-100.16	-316.97
	298	2.211	-5.478		-317.72
	303	1.636	-4.121		-316.96

References

1. Rudzinski, W.; Plazinski, W. Kinetics of solute adsorption at solid/solution interfaces: A theoretical development of the empirical pseudo-first and pseudo-second order kinetic rate equations, based on applying the statistical rate theory of interfacial transport. *J. Phys. Chem.* **2006**, *110*, 16514-16525.

2. Bulut, Y.; Aydın, H. A kinetics and thermodynamics study of methylene blue adsorption on wheat shells. *Desalination.* **2006**, *194*, 259-267.

3. Zhang, X.; Bai, R., Mechanisms and kinetics of humic acid adsorption onto chitosan-coated granules. *J. Colloid. Interf Sci.* **2003**, *264*, 30-38.

4. Peng, Q.; Liu, Y.; Zeng, G.; Xu, W.; Yang, C.; Zhang, J., Biosorption of copper(II) by immobilizing Saccharomyces cerevisiae on the surface of chitosan-coated magnetic nanoparticles from aqueous solution. *J. Hazard. Mater*. **2010**, *177*, 676-682.

5. Günay, A.; Arslankaya, E.; Tosun, İ., Lead removal from aqueous solution by natural and pretreated clinoptilolite: Adsorption equilibrium and kinetics. *J. Hazard. Mater.* **2007**, *146*, 362-371.
